# Supplementary material for: A mathematical model coupling polarity signaling to cell adhesion explains diverse cell migration patterns
Source: PLoS Comput Biol. 2017 May 4;13(5):e1005524. doi: 10.1371/journal.pcbi.1005524 (PMC5436877; doi:10.1371/journal.pcbi.1005524)
Supplement: S1 Text — (PDF) [file pcbi.1005524.s001.pdf]

# S1 TEXT FOR: A MATHEMATICAL MODEL COUPLING POLARITY SIGNALING TO CELL ADHESION EXPLAINS DIVERSE CELL MIGRATION PATTERNS

WILLIAM R. HOLMES

*Department of Physics and Astronomy, Vanderbilt University*

JINSEOK PARK AND ANDRE LEVCHENKO

*Department of Biomedical Engineering, Yale University*

LEAH EDELSTEIN-KESHET

*Department of Mathematics, University of British Columbia, Vancouver BC, Canada*

## 1. Methods

We simulate models using MatLab's (MathWorks, Natick MA) built-in ODE library (ODE45). We use dynamical systems bifurcation techniques and the package Matcont [1] to map the parameter space of each model and create bifurcation diagrams. In each case, we chose a basic set of parameters to achieve bistability in the absence of oscillatory feedback. Feedback was then introduced and parameters were varied to determine whether a given model variant is consistent with experimental manipulations described by [2].

## 2. Model equations

2.1. **Model 1, ECM signaling equation analysis.** Consider the ECM signaling equations from the maintext with $A_E(R_{1,2}), L_E(\rho_{1,2})$ taken to be parameters. The equations for the ECM are then

$$\frac{dE_k}{dt} = \epsilon\left[(B_E + A_E E_k) - E_k\left(L_E E_k + l_c E_j\right)\right], \quad j \neq k.$$

This is the Lotka-Volterra species-competition model, briefly analyzed here. This system admits four possible solutions $s_1 = (0,0)$, $s_2 = (0, E_2^*)$, $s_3 = (E_1^*, 0)$, and $s_4 = (E_1^{**}, E_2^{**})$ where

$$E_{1,2}^* = \frac{A_E(R_{1,2})}{L_E(\rho_{1,2})}, \qquad \text{and} \qquad E_{1,2}^{**} = \frac{A_E(R_{1,2})L_E(\rho_{2,1}) - A_E(\rho_{2,1})l_c}{L_E(\rho_{1,2})L_E(\rho_{2,1}) - l_c^2}. \tag{2.1}$$

*Date*: April 24, 2017.

Then $s_{2,3}$ are associated with two polarized states while $s_4$ is an apolar co-existence state. In region I, the only stable solution is $s_4$. In regions II and III, solutions $s_2$ and $s_3$ respectively are stable. In Regime IV, only $s_1$ is stable.

2.2. **Model 1a.** Using $R_I = \rho_I = 1$ as before, and taking $n = 1$, $B_E = 0$ and a linear assumption for $b_\rho$, we arrive at the model equations

$$\frac{dR_k}{dt} = \frac{b_R}{1+\rho_k} R_I - \delta R_k, \tag{2.2a}$$

$$\frac{d\rho_k}{dt} = \frac{(k_E + \bar{\gamma}_E E_k)}{1+R_k} \rho_I - \rho_k, \tag{2.2b}$$

$$\frac{dE_k}{dt} = \epsilon \left[ (k_R + \bar{\gamma}_R R_k)E_k - ((k_\rho + \bar{\gamma}_\rho \rho_k)E_k^2 + l_c E_j E_k) \right], \tag{2.2c}$$

for $k = 1, 2$ representing quantities in the two lamellipods and $j \neq k$ representing quantities in the other.

2.3. **Model 1b.** The equations for Model 1b are the same as those of Model 1a, but with conservation of total GTPases, $R_I = R_T - R_1 - R_2$ and $\rho_I = \rho_T - \rho_1 - \rho_2$.

Technically, the transition from polarized to oscillatory behavior shown in Figure 3 results from a bifurcation to heteroclinic cycles rather than a canonical Hopf bifurcation. (But the conclusions are similar.) In the oscillatory regime of this model, there are two unstable polarized states. The oscillation is a heteroclinic cycle connecting these two states. As the bifurcation is approached from the oscillatory side, the period of oscillation becomes infinitely long, and at the bifurcation the system spends infinite time at one or the other polarized states, representing the transition to stability.

2.3.1. *Model 1b parameter set:* $k_R = 1, l_c = 1, k_\rho = 0.1, k_E = 0, \bar{\gamma}_E = 5, \epsilon = 10, b_R = 0.5, \delta = 1, \bar{\gamma}_R = 0.75, \bar{\gamma}_\rho = 1, n = 1, R_T = \rho_T = 1.$

2.4. **Model 1c.** This model augments Model 1b to consider the influence of Rac and Rho effector molecules in the protrusion and contraction process. Here $w_k$ represents effectors of Rac and $c_k$ effectors of Rho in lamellipod $k$. We assume that these directly influence protrusion and contraction terms ($B_E$ and $L_E$) and that their dynamics are described by linear ODE's. We retain the same Rac and Rho equations as in Model 1b and include the following to describe dynamics of $E_k, w_k, c_k$.

$$\frac{dE_k}{dt} = \epsilon \left[ (k_R + w_k)E_k - ((k_\rho + c_k)E_k^2 + \beta E_j E_k) \right], \quad j \neq k, \tag{2.3a}$$

$$\frac{dw_k}{dt} = \epsilon_2(\gamma_R R_k - w_k), \tag{2.3b}$$

$$\frac{dc_k}{dt} = \epsilon_2(\gamma_\rho \rho_k - c_k). \tag{2.3c}$$

Here, $1/\epsilon_2$ is a timescale variable associated with the speed of these reactions. Small $\epsilon_2$ indicates $w_k$ and $c_k$ are slow variables.

This reformulation leads to three explicit timescales representing GTPase dynamics $O(1)$, dynamics of GTPase effectors $O(\epsilon_2)$, and dynamics of the ECM $O(\epsilon)$. Model **1**c (Eqs. (2.3))

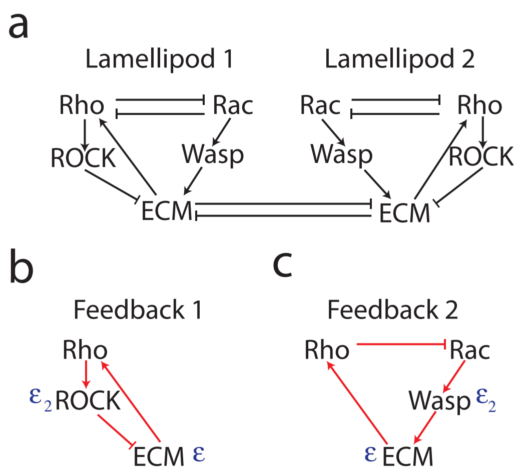


FIGURE A. Schematic diagram for Model **1**c. Similar to Figure 2a except with the explicit inclusion of intermediate effectors such as Rock and Wasp / Wave. **(a)** Full model describing the proposed interactions between the ECM and GTPase signalling. **(b,c)** Diagram of the two negative feedback loops embedded in this model that are capable of producing oscillations. $\epsilon_1, \epsilon_2, \epsilon_3$ are rate constants for GTPase dynamics, Rock / Wasp dynamics, and ECM dynamics respectively, see (2.3).

asymptotically reduces to Model **1**b provided the GTPase effector dynamics (e.g. ROCK / WASP) are fast ($\epsilon_2 \gg \epsilon$). In this case a quasi steady state approximation reduces Model **1**c to Model **1**b and bifurcation analysis of the full Model **1**c equations (not shown) closely matches that of Figure 3. In that case, Model 1c has the same problem that GTPase dynamics are required to be slow to generate oscillations. When $\epsilon_2 \ll \epsilon$, the effectors determine the timescale of feedbacks, and consequently, GTPases need not be slower than ECM dynamics (e.g. it can be the case that $\epsilon < 1$).

## 3. Model 2

We set $R_I = \rho_I = 1$, and Hill coefficients $n = 3$, and use the assumed $b_\rho(E_k)$ expression. Setting $A_E = 0$, and using assumed forms of $B_E$ and $L_E$, we get (for $k = 1, 2$ and $j \neq k$):

$$\frac{dR_k}{dt} = \frac{b_R}{1+\rho_k^3} R_I - \delta R_k, \tag{3.1a}$$

$$\frac{d\rho_k}{dt} = \left(k_E + \gamma_E \frac{E_k^3}{E_0^3 + E_k^3}\right) \frac{1}{1+R_k^3} \rho_I - \rho_k, \tag{3.1b}$$

$$\frac{dE_k}{dt} = \epsilon \left[\left(k_R + \gamma_R \frac{R_k^3}{R_0^3 + R_k^3}\right) - E_k^2 \left(k_\rho + \gamma_\rho \frac{\rho_k^3}{\rho_0^3 + \rho_k^3}\right) - l_c E_j E_k\right]. \tag{3.1c}$$

3.0.1. *Model 2 parameter set.* $b_R = 2, n = 3, \delta = 1, k_E = k_R = k_\rho = 0, \gamma_E = 5, R_0 = 0.85, \rho_0 = 0.85, E_0 = 1, l_c = 1, \epsilon = 0.1, R_I = \rho_I = 1$.

## 4. Model 3

We use the conservation of GTPases combined with the bistable GTPase model (2) to obtain the equations:

$$\frac{dR_k}{dt} = \frac{b_R}{1+\rho_k^3}(R_T - R_1 - R_2) - \delta R_k, \tag{4.1a}$$

$$\frac{d\rho_k}{dt} = \left(k_E + \gamma_E \frac{E_k^3}{E_0^3 + E_k^3}\right) \frac{1}{1+R_k^3}(\rho_T - \rho_1 - \rho_2) - \rho_k. \tag{4.1b}$$

$$\frac{dE_k}{dt} = \epsilon \left[\left(k_R + \gamma_R \frac{R_k^3}{R_0^3 + R_k^3}\right) - E_k^2 \left(k_\rho + \gamma_\rho \frac{\rho_k^3}{\rho_0^3 + \rho_k^3}\right) - l_c E_j E_k\right]. \tag{4.1c}$$

4.0.1. *Model 3 parameter set.* The base parameters used for this model are $R_T = \rho_T = 2, \delta = 1, b_R = 5, R_0 = \rho_0 = 0.85, E_0 = 1, n = 3, k_E = 3, \gamma_E = 2, \epsilon = 0.1, k_\rho = 0.2, k_R = 0.2, \gamma_R = 0.3, l_c = 0$.

4.1. **Model 3b.** In Figure 5d, we demonstrate the influence of ECM feedback on Rac activation. To do so, we consider a modification of Model 3 given by:

$$\frac{dR_k}{dt} = \left(b_R + \gamma_{ER} \frac{E_k^3}{E_0^3 + E_k^3}\right) \frac{1}{1+\rho_k^3}(R_T - R_1 - R_2) - \delta R_k, \tag{4.2a}$$

$$\frac{d\rho_k}{dt} = \left(k_E + \gamma_E \frac{E_k^3}{E_0^3 + E_k^3}\right) \frac{1}{1+R_k^3}(\rho_T - \rho_1 - \rho_2) - \rho_k. \tag{4.2b}$$

$$\frac{dE_k}{dt} = \epsilon \left[\left(k_R + \gamma_R \frac{R_k^3}{R_0^3 + R_k^3}\right) - E_k^2 \left(k_\rho + \gamma_\rho \frac{\rho_k^3}{\rho_0^3 + \rho_k^3}\right) - l_c E_j E_k\right]. \tag{4.2c}$$

Here, the parameter $\gamma_{ER}$ represents the magnitude of ECM feedback on the rate of Rac activation. Setting $\gamma_{ER} = 0$, reduces this variant to the original Model 3.

4.1.1. *Model 3b parameter set.* All parameters are the same as in Model 3 with $\gamma_{ER} = 0.5$. This parameter set was used to generate the dashed borders in Figure 5d. Note that the strength of ECM feedback to Rho activation is $\gamma_E = 2$. This parameter set represents a scenario where ECM influences both Rac and Rho, but where the influence on Rho is dominant.

## 5. Parameter Selection

In our models, a slow negative feedback interacts with a bistable system to generate a relaxation oscillator. This suggests a strategy to find appropriate parameter regimes: first pick parameters for the bistable submodel that provide a reasonable range of behavior (spanning the low monostable, bistable, and high monostable states); then parametrize the negative feedback subsystem so that the full system swings across those three regimes.

For example, in Model 3, bistability stems from the conserved GTPase dynamics. Our experience with such models stems from both dimension-carrying versions [3, 4], and from a theoretical analysis of the Rac-Rho system [5]. To first decouple the ECM dynamics,

we considered $b_\rho$ as a fixed parameter. We reduce the dimensionality of parameter space by scaling the variables. Here we scaled time by the typical GTPase inactivation time $1/\delta$ (equivalent to setting $\delta = 1$ in the full model) and GTPase concentration by the "IC50" level (at which GTPase rate of activation is inhibited down to 50% of its maximal level. (This is equivalent to setting the Hill function IC50 parameters to 1.) Past experience suggests that the system exhibits bistability when the total amount of GTPases is roughly double the value of the IC50 parameter. Thus we chose the dimensionless total concentration of GTPase to be $R_T = \rho_T = 2$. Finally, we selected $b_R$ and the range of $b_\rho$ to span the bistable domain (as noted above) using bifurcation analysis of the Rac / Rho system (Figure 5a). This fully parameterized the GTPase submodel.

We next parameterized the negative feedback subsystem so that the resulting range of values of $E_i$ (max and min) suffice to force the parameter $b_\rho(E_i)$, now considered a function of $E_i$, to traverse the bistable region in Figure 5a. To do so, $k_E$ and $\gamma_E$ were chosen to determine the maximum and minimum values of $b_\rho$. We scaled the ECM variables by the Hill function parameter $E_0$ (level of ECM that leads to half-maximal ECM-feedback-induced Rho activation rate). Then the ECM feedback in Eqn. (4.1c) switches on and off in the proximity of $E_j \approx 1$, determining the required range of interest for the variables $E_j$ to ensure $b_\rho(E_i)$ spans the range $[k_E, k_E + \gamma_E]$. The maximum and minimum values of $E_i$ are related to the quantities $(k_R + \gamma_R)/k_\rho$ and $k_R/(k_\rho + \gamma_\rho)$. These are set to ensure that $E_j$'s operate in a reasonable range about the "switching value" of 1. The Hill parameters $R_0$ and $\rho_0$ (half-max levels of Rac and Rho for their respective effects on ECM in Eqn. (4.1b)) were adjusted with the same goal in mind, to ensure that $(E_j)$ is able to nearly achieve their required maximum and minimum values. Here we also used the behavior of the decoupled bistable subsystem, namely the maximum and minimum values for Rac and Rho in the two steady states. The midpoint, (max-min)/2, was a reasonable guess for appropriate values of $R_0$ and $\rho_0$. Finally, some small adjustments were made to this basic parameter set to obtain the final results. With this basic simple strategy it was easy to find large parameter swaths that produced the three regimes of behavior and the models were robust to parameter variations.

## References


[1] Dhooge A, Govaerts W, Kuznetsov YA (2003) Matcont: A Matlab package for numerical bifurcation analysis of ODEs. ACM TOMS 29: 141–164.

[2] Park J, Holmes WR, Lee SH, Kim HN, Kim DH, et al. (2017) A mechano-chemical feedback underlies co-existence of qualitatively distinct cell polarity patterns within diverse cell populations. arXiv:161208948v1 .

[3] Marée AF, Jilkine A, Dawes A, Grieneisen VA, Edelstein-Keshet L (2006) Polarization and movement of keratocytes: A multiscale modelling approach. Bull Math Biol 68: 1169-1211.

[4] Jilkine A, Marée AF, Edelstein-Keshet L (2007) Mathematical model for spatial segregation of the Rho-Family GTPases based on inhibitory crosstalk. Bull Math Biol 69: 1943-1978.

[5] Holmes WR, Edelstein-Keshet L (2016) Analysis of a minimal Rho-GTPase circuit regulating cell shape. Physical Biology 13: 046001.
